# Family social environment in childhood and self-rated health in young adulthood

**DOI:** 10.1186/1471-2458-11-949

**Published:** 2011-12-22

**Authors:** Christelle Roustit, Eric Campoy, Emilie Renahy, Gary King, Isabelle Parizot, Pierre Chauvin

**Affiliations:** 1 INSERM, U707, Research Group on the Social Determinants of Health and Healthcare, Paris 75012, France; 2 Univ. Paris 06, UMR S 707, Paris 75012, France; 3 Univ. Paris-Dauphine, DRM, CREPA (CNRS, UMR 7088), Paris 75016, France; 4 Dept Epidemiology, Biostatistics & Occupational Health, International Research Infrastructure on Social inequalities in health (IRIS), McGill University, Montreal, QC, Canada; 5 Department of Biobehavioral Health, Pennsylvania State University, University Park, PA, USA; 6 CNRS, UMR 8097, Centre Maurice Halbwachs, Paris 75014, France; 7 Department of Public Health, AP-HP, Hôpital Saint Antoine, Paris 75012, France

## Abstract

**Background:**

Family social support, as a form of social capital, contributes to social health disparities at different age of life. In a life-course epidemiological perspective, the aims of our study were to examine the association between self-reported family social environment during childhood and self-reported health in young adulthood and to assess the role of family functioning during childhood as a potential mediating factor in explaining the association between family breakup in childhood and self-reported health in young adulthood.

**Methods:**

We analyzed data from the first wave of the Health, Inequalities and Social Ruptures Survey (SIRS), a longitudinal health and socio-epidemiological survey of a random sample of 3000 households initiated in the Paris metropolitan area in 2005. Sample-weighted logistic regression analyses were performed to determine the association between the quality of family social environment in childhood and self-rated health (overall health, physical health and psychological well-being) in young adults (n = 1006). We used structural equation model to explore the mediating role of the quality of family functioning in childhood in the association between family breakup in childhood and self-rated health in young adulthood.

**Results:**

The multivariate results support an association between a negative family social environment in childhood and poor self-perceived health in adulthood. The association found between parental separation or divorce in childhood and poor self-perceived health in adulthood was mediated by parent-child relationships and by having witnessed interparental violence during childhood.

**Conclusion:**

These results argue for interventions that enhance family cohesion, particularly after family disruptions during childhood, to promote health in young adulthood.

## Background

Research studies using measures of social stratification based on the three core dimensions of socioeconomic status, namely, level of education, occupational status and income, social health inequalities are being increasingly explored through the life-course epidemiological approach [1-5]. Adverse life events or circumstances can constitute pathways between social conditions and disparities in health outcomes (pathway model) or lead to social inequalities in economic resources, which in turn, are associated with poor health outcomes (i.e., accumulation model) [6]. Research studies in this field are contributing to the health inequality debate, thus providing data for policy makers to allocate human resources to disadvantaged families with children as a means of improving population health or to increase financial resources for families through public policies [7,8].

In this research field, the family social environment that includes emotional and social support has been documented as one of the predictors of numerous health outcomes in childhood and adulthood [9,10]. The quality of family functioning, including emotional support, nonviolent interparental relationships and parents'emotional stability, has been shown to be associated with a significantly lower risk of psychosocial maladjustment in children and adolescents [11,12]. In the 35-year follow-up of the Harvard Mastery of Stress Study, Russek and Schwartz showed that the subjects who gave lower scores for "perceived caring" items while in college had coronary artery disease, hypertension, duodenal ulcer and alcoholism in midlife [13]. Yet, one of the critical situations where the family social environment and family cohesion are impaired is parental separation or divorce, which has increased over the last 40 years in industrialized countries. In the United States, the divorce rate was 3.6 per 1000 population (i.e., a total of 1.07 million divorces) in 2005, which is still one of the highest in the world [14]. In OECD (Organisation for Economic Co-operation and Development) countries, the crude divorce rate was 2.2 per 1000 population in 2001, and the number of divorces was 44.4 per 100 marriages in 2002. In France, divorces increased from 38.2 per 100 marriages in 1995-52.2 per 100 marriages in 2005[15]. These changes in family structure have been shown to have a significant impact on the well-being of children [16-18] and, later, on young adults in terms of psychosocial adjustment and social integration in adult life [19-25]. In a meta-analysis, Amato [26,27] found that children from divorced families experience higher levels of behavioural, psychological and cognitive development impairments than children from intact families. The short-term consequences include lower levels of scholastic performance [28] and a higher risk of psychosocial maladjustment, such as depression, conduct disorders, psychoactive substance use [29-31] and difficulties in relationships with family and peers. In a review of long-term studies, Wallerstein [32] reported that children from single parent families are more likely to divorce during their own adulthood than children raised in intact families. With regard to the dimensions of psychological well-being such as happiness, self-rated health and self-satisfaction in social life, and risky health behaviours and interpersonal relationships, children from disrupted families are more likely to be on the negative end of the rating scale [33,34]. More recently, the Hunt-study II, a Norwegian longitudinal study showed a higher risk of emotional disturbances and scholastic difficulties in adolescents from disrupted families [35,36].

In the case of family breakup in childhood, several theoretical perspectives have been posited to explain the link between the family social environment and short and long-term health consequences [37]. The family stress model [38] assumes that parenting competence is impeded by parental psychological distress due to marital separation or economic difficulties. Children of separated parents with psychological distress are exposed to insecure marital attachment and marital conflict and come to feel that their own well-being is threatened. Accordingly, family-centred approaches aim to alleviate parental distress and to serve as a buffer in the relationship between the child and the custodial parent [39]. The investment theory argues that economic disadvantages negatively impact education and leisure spending that contribute to a child's well-being [40]. Indeed, parental separation leads to a possible dramatic decline in the standard of living in the custodial household. In social epidemiology, the life-course approach has been employed to demonstrate the association between material deprivation in childhood and further health inequalities in adulthood [41-43] On the other hand; Foster and Kalil showed that cognitive and behavioural outcomes did not vary by family structure among economically disadvantaged pre-schoolers, irrespective of race-ethnicity [44]. Therefore, the impact of social policies for single parent households in the last decade on the association between parental separation and psychosocial maladjustment in adolescence and later in adulthood remains to be demonstrated in the next years [45].

In this context, investigating mediational pathways between parental separation or divorce in childhood and future self-perceived health could be a useful approach for promoting health in young adulthood. Although many studies have reported protective factors in pediatric populations, such as temperament, coping styles, family process factors, such as low-conflict and positive parenting, and supportive relationships with peers [24,35] only a few of them have tested these factors as potential mediators of the association between family breakup in childhood and health status in young adulthood. Specifically, the goal of our study was to analyze the association between parental divorce or separation before 18 years old and adult self-reported health status and to examine the mediating role of self-reported family functioning in childhood.

## Methods

### Subjects and database

This study was based on data collected from the Health, Inequalities and Social Ruptures cohort study (SIRS [Santé, Inégalités et Ruptures Sociales]), a longitudinal health and epidemiological survey of the general adult population of the Paris metropolitan area (Paris and its suburbs, a region with a population of 6.5 million). The data collection [46] was initiated in 2005 and conducted by the National Institute of Health and Medical Research (INSERM) as part of a collaborative research project with the National Centre for Scientific Research (CNRS) and the National Institute for Demographic Studies (INED). The study was approved by France's privacy and personal data protection authority (Commission Nationale de l'Informatique et des Libertés). The first wave of data collection was carried out between September and December, 2005. The study was based on a 3-stage cluster random sample of 4560 adults stratified according to the neighborhood socioeconomic status. The primary sampling units were census blocks called "IRISs" ("IRIS" is a French acronym for blocks for incorporating statistical information). They constitute the smallest census unit areas in France (with about 2000 inhabitants each) whose aggregate data can be used on a routine basis. The Paris metropolitan area was divided into six strata according to the population's socioeconomic profile [47] to oversample the poorest neighbourhoods, and census blocks were randomly chosen within each stratum. In all, 50 census blocks were selected from the 2595 eligible census blocks in Paris and its suburbs. Subsequently, within each selected census block, 60 households were randomly chosen from a complete list of households. Lastly, one adult was randomly selected from each household by the birthday method.

Among the respondents, 3.1% who did not speak French and 1.8% who were too sick to answer the questions were excluded from the study population, and 28.6% refused to participate. Overall, 3023 subjects were included in the cohort and completed the first-wave of interviews. Data on health and health-related characteristics, familial and social functioning, and past life-events were collected through at-home through face-to-face interviews. Retrospective data on childhood circumstances and cross-sectional data on adulthood indicators collected during the first wave of data collection were employed to examine the effect of parental separation during the first stages of the adult life cycle. Thus, we selected the first tertile-1006 young adults aged 18-37 years old-of the age distribution of the surveyed population.

### Indicators and variables

*Self-rated health*, the dependent variable, was measured on a five-point Likert scale (1 = very good, 2 = good, 3 = fair, 4 = poor and 5 = very poor), based on the question "Would you say that your health in general is very good, good, fair, poor or very poor?" It was supplemented by the following two questions: "How would you rate your physical health?" (1 = very good, 2 = good, 3 = fair, 4 = poor and 5 = very poor) and "How would you rate your psychological health and emotional well-being?" (1 = very good, 2 = good, 3 = fair, 4 = poor and 5 = very poor). Each indicator of self-reported health was coded according to a trichotomous outcome variable (0 = very good, 1 = good, 2 = fair, poor or very poor). According to Idler et al. [48], self-reported health is a valid predictor of mortality, and this indicator is highly correlated with chronic health problems, functioning status, and health services utilization [49]. Despite a high correlation between the items defining perceived health, whether general health, physical health or psychological health (Cronbach's Alpha = 0.84), we chose not to combine the three health items into one summary score.

#### Family functioning during childhood

Measures of family functioning during childhood were incorporated based on previous studies showing the relationships between parental conflict, parent-child hostility and psychosocial maladjustment in childhood [11] and later in adulthood [50]. Data on these characteristics were the *perceived quality of parent-child relationships *and *exposure to interparental violence in childhood*. The quality of maternal and paternal relationships was assessed separately with the following question: "Before age 18, how were your relationships with your mother/father?" (1 = very good, 2 = good, 3 = poor, 4 = very poor, 5 = no relationship, 6 = sometimes good, sometimes bad, 7 = other). The answer was coded as a dichotomous outcome variable (1 = good relationships for very good, good, sometimes good or sometimes bad; 2 = poor relationships for poor, very poor or no relationship; and "7" responses were excluded). Evidence of interparental violence in childhood before age 18 was determined by the following question: "Did you witness interparental violence before the age of 18?" (Yes/No). The three familial items were combined to create a single score from 0 (excellent family functioning prior to age 18) to 3 (very poor family functioning prior to age 18). Family breakup in childhood before age 18 was determined by the following question: "Did your parents separate before you were 18 years old?" (Yes/No).

In addition to age and gender, we adjusted for socioeconomic factors in adulthood recorded at the time of the survey. Individual socioeconomic status (SES) was assessed on the basis of three variables: education, monthly household income per consumer unit, and occupational status. Education was measured by a 3-level hierarchical variable: primary, secondary, postsecondary or university. Monthly household income per consumer unit was self-reported and ranked based on the quartile values of its distribution in the study population. Occupational status was assessed according to the French classification of professions and social categories [Catégories Socio-Professionnelles] (never-active; upper white collar; middle and lower white collar; blue collar; saleswomen, craftswomen and managers grouped together). Lastly, we adjusted for marital status (0 = single, 1 = couple).

### Statistical analysis

The first step of the analysis consisted of examining the bivariate association between the family functioning variables and family breakup in childhood, and the three health status outcomes (dependent variables). Second, using adjusted regression models, we tested the associations between the occurrence of family breakup and the family functioning variables.

Lastly, we used structural equation modelling to test for mediation. The variables significantly associated with family breakup in Step 2 were introduced into a path analysis. We compared the direct and indirect effect of family breakup on self-reported health variables after introducing these variables into the models as mediators in the association between family breakup and self-rated health. The following fit indices were used to assess the model fit: the root-mean square error of approximation (RMSEA) and the normed-fit index (NFI). Statistical significance was defined as a p < 0.05. SPSS^® ^19.0 and EQS^® ^6.1 were respectively used to perform logistic regression analyses and structural equation models.

## Results

Of the 1006 young people aged 18-37 years old who completed the questionnaire, 13.6% reported a fair, poor, or very poor general health; 16.3%, a fair, poor, or very poor physical health; and 20.3%, a fair, poor, or very poor psychological or emotional health.

Respondents from disrupted families in childhood or reporting poorer family functioning during childhood ranked more at the negative end of the health scales (Table 1).

**Table 1:** Comparison of family characteristics during childhood by self-perceived health in adulthood (N = 1006)

	N	General health				Physical health				Psychological health			
		
		Very good	Good	Fair, poor or very poor	*p-value*	Very good	Good	Fair, poor or very poor	*p-value*	Very good	Good	Fair, poor or very poor	*p-value*
*Demographics*													
Age, mean	1006	27.4	28.2	27.3	*.020*	27.4	28.0	28.0	*.124*	27.2	28.0	28.4	*.010*
Female, %	1006	52.2	47.7	66.7	*.001*	50.1	50.0	35.3	*.003*	46.9	53.2	60.1	*.007*
*Family characteristics during childhood *													
Poor maternal relationships, %	1001	8.3	10.4	25.0	*< .001*	8.5	10.7	20.8	*< .001*	7.1	11.0	20.9	*< .001*
Poor paternal relationships, %	1004	20.0	28.4	38.6	*< .001*	19.1	29.5	32.7	*< .001*	15.7	29.3	40.1	*< .001*
Interparental violence, %	991	11.8	21.3	27.1	*< .001*	13.3	19.2	24.7	*.003*	12.2	17.4	30.9	*< .001*
Parental divorce or separation, %	1006	16.8	20.8	28.8	*.007*	15.9	21.3	26.7	*.009*	16.6	20.7	25.3	*.036*

Table 2 shows changes in the odds ratios for the independent association between family variables during childhood (family breakup and family functioning variables) and self-rated health. Results support the association between family disruption in childhood and poor self-perceived general health status (OR = 1.93 [1.16-3.20]), poor self-rated physical health (OR = 1.98 [1.19-3.28]) and poor psychological well-being (OR = 1.79 [1.12-2.84]). These associations were adjusted for age, gender, marital status, and socioeconomic variables in adulthood. Each variable defining the quality of family functioning in childhood (maternal or paternal relationships and witnessing interparental violence) were also independently associated with perceived health in adulthood.

**Table 2:** Bivariate logistic regression: Adjusted odds ratios and 95% confidence intervals for the association between family characteristics during childhood and self-perceived health in adulthood

		General health status (Ref.:very good)		Physical health (Ref.:very good)		Psychological health (Ref.:very good)	
		
	N	Very poor/poor/fair	Good	Very poor/poor/fair	Good	Very poor/poor/fair	Good
***Family characteristics during childhood ***							
Separation or divorce	1006	1.93* (1.16-3.20)	1.36 (0.95-1.94)	1.98** (1.19-3.28)	1.65** (1.14-2.40)	1.79* (1.12-2.84)	1.52* (1.04-2.22)
Poor maternal relationships	1001	3.81*** (2.19-6.65)	1.31 (0.83-2.05)	2.87*** (1.63-5.06)	1.25 (0.79-1.98)	4.03*** (2.33-6.99)	1.72* (1.04-2.84)
Poor paternal relationships	1004	2.42*** (1.56-3.76)	1.55** (1.15-2.09)	2.05** (1.32-3.19)	1.78*** (1.30-2.43)	3.63*** (2.42-5.45)	2.25*** (1.62-3.14)
Interparental violence	991	2.83*** (1.71-4.67)	1.95*** (1.37-2.77)	2.33** (1.43-3.80)	1.42 (0.99-2.04)	3.31*** (2.13-5.14)	1.53* (1.05-2.24)

^a^Adjusted for age, gender, marital status and current socioeconomic status

*CI *= confidence interval

*P < .05; **P < .01; ***P < .001

To find a potential mediation in our model between family breakup and self-perceived health, the associations were tested between exposure to parental breakup in childhood and family functioning variables during childhood adjusted for age, gender, marital status and socioeconomic status in adult life (Table 3). Having experienced parental separation or divorce was significantly associated with poor paternal relationships (OR = 3.36;CI = [2.44-4.63]) and/or poor maternal relationships (OR = 2.11;CI = [1.37-3.24]) and with having witnessed interparental violence (OR = 5.05;CI = [3.57-7.14]).

**Table 3:** Bivariate logistic regression: Adjusted odds ratios and 95% confidence intervals for associations between family breakup in childhood and family functioning variables as outcomes^a^

		Parental divorce or separation before age 18	
*Outcomes*	N	Odds Ratio	95% CI
***Family functioning indicators***			
Poor maternal relationships	1001	2.11***	1.37-3.24
Poor paternal relationships	1004	3.36***	2.44-4.63
Interparental violence	991	5.05***	3.57-7.14

^a^Controlling for gender, age, marital status and current socioeconomic status

*CI *= confidence interval***P < .001

**Figure 1:**
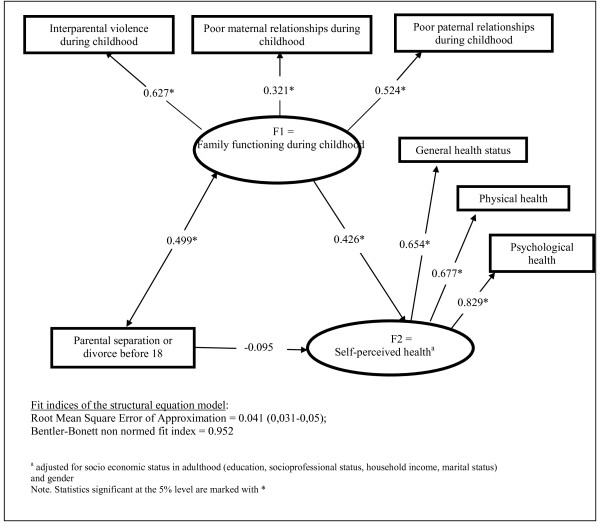
**Structural Equation Model**. Mediating model of the association between parental separation or divorce before 18 and self-perceived health.

A path diagram representing the SEM results is shown in Figure 1. Family functioning during childhood and self-rated health were modelled as latent variables. An examination of the path coefficients shows that the occurrence of parental separation or divorce before 18 was significantly associated with the latent variable defining the poorer quality of family functioning (β = 0.499, *p *≤ 0.001), which was also associated with poorer self-perceived health status (β = 0.426, *p *≤ 0.001). The indirect standardized effect of parental separation or divorce before 18 on self perceived health in adulthood was 0.21 (0.499 × 0.426; *p *≤ 0.001), whereas direct effect was -0.095 (*p *> 0.05). In a model without mediational variables--such as family functioning during childhood-(figure not shown), the direct standardized effect of parental separation or divorce before 18 years old on self perceived health in adulthood was estimated to be 0.12 (*p *≤ 0.001). Therefore, the percentage of parental separation (or divorce before 18) effect mediated by family functioning variables in childhood was 63% (= 0.21/[0.21 + 0.12]). Overall model fit appears quite good: the root mean square error of approximation was within the acceptable range (RMSEA = 0.041 [0.031-0.05]) and the Bentler-Bonett non-normed fit index was 0.952.

## Discussion

Our first objective was to examine the association between the family social environment before age 18 years old and self-perceived health outcomes in young adulthood. This study was conducted among a representative sample of the Paris metropolitan area and is the first study of its kind in France. Of primary note, is the association found between the quality of the family social environment in childhood and self-rated health in adulthood. In addition, the analysis revealed that 63% of the effect of parental separation on self-rated health in young adulthood is due to the quality of the family functioning in childhood. In our study, the parental separation (or divorce before 18) is deleterious for self-rated health in adulthood only because of family violence and child-parents relationships.

These findings are consistent with other studies demonstrating the effect of earlier familial relationships on later health outcomes. Previous research showed that parental emotional support, interparental violence and parental distress were related to and mediated the association between family breakup in childhood and psychosocial maladjustment in adolescence [31].

These findings support the life-course continuity proposition of exposure to relational disadvantages at an earlier age and negative health outcomes at different developmental stages during adolescence and young adulthood. Exposure to a risky family environment is associated with many different factors, which can modify the individual's life trajectory. For example, research studies have shown that many forms of childhood adversities due to parental separation are associated with an increased risk of depression in adolescence and early adulthood [51-54] and that early-onset depression is associated with a high recurrence risk. Depression or other childhood or adolescent psychopathologies can occur after parental separation and serve as a pathway between family breakup and adult mood disorders, [55] which can lower health ratings.

The second aim of this paper was to explore the mediating role of the family functioning variables in the association between family breakup in childhood and health status outcomes in adulthood. Our findings are consistent with the psychosocial perspective [33] and the mediational model supports related hypothetical mechanisms underlying the association between parental separation in childhood and health status in adulthood. In this connection, parent-child transactional processes are essential for ensuring cohesion, upon which the children's identity builds,[56] and continuity in the adaptation to different developmental tasks[57,58]. This first framework can be supplemented with a behavioural model through a social approach. The family is the first level of social support. It lays the foundation for the social network, which promotes growth of the capacity for experimentation, autonomy and acquiring independence during adolescence and young adulthood so that the child can respond actively to life's challenges [59]. Social relations, in particular, in families, influence individual behaviour by shaping norms around healthy attitudes and by using collective prevention resources [60]. As well, social networks provide social support, which influences physical and psychological health [61]. Parental separation triggers a period of family relationship reorganization and the renegotiation of the family's boundaries [62]. Those exposed to interparental violence often have threatened self-esteem, which is associated with depression and impaired social functioning [63-65]. Consequently, any impairment in social support due to overt conflict and hostility in families may have an impact on health status at older ages.

However, our study had several limitations. The main weakness of the analysis is the retrospective reporting of family variables (separation before age 18, family dysfunction and violence). It's difficult to determine the timing of each of these entangled variables which limits one's ability to interpret the path/mediation analyses done using structural equation models. The retrospective study design may have induced some recall bias. Current circumstances, social life or depressed mood can lead to a significant increase in reports of dysfunctional relationships with parents in childhood [66]. In the literature, the analysis of the validity of retrospective studies has shown that the prevalence of past events is underestimated [67]. But adverse childhood experiences can also be recalled to a greater degree by psychologically impaired individuals as a result of internal biographical coherence [68]. In particular, recalling domestic violence upon a single question may have been connoted by the subject's emotional state during the face-to-face interview, which, in the more psychologically fragile subjects, would have induced same-source bias.

Secondly, because of the cross-sectional nature of the data, we could not suggest causality between family breakup and family functioning. Family breakups are, to a large extent, detrimental to children because of the atmosphere in which the separation occurs and not merely because of the resulting quality of the interrelationships. Thirdly, we adjusted only for the current family structure and the usual socioeconomic variables but did not take into account all the other stressful events during childhood or in adult life (such as divorce or separation). Yet, marital instability is highly associated with both parental separation and negative health outcomes. Lastly, we did not introduce information about the atmosphere in which the separation occurred or the resulting quality of the interparental relationship, both of which could affect children.

## Conclusion

This study sheds light on the mechanisms that mediate the relationship between family separation in childhood and health status in young adulthood. Our findings are consistent with the public health implications of family disruption in terms of interventions at three levels: familial (clinical support, family centred-approach), legal (mediation, joint custody), and micro-and macro-social. Additional research is thus needed to evaluate these interventions in the field of health promotion. Indeed, envisioning a healthy future for our children prompts us to pay close attention to the model through which they interact with their social environment. Doing this requires focusing both on the family constellation that we create for our children and the material conditions that we provide to them.

## Competing interests

The authors declare that they have no competing interests.

## Authors' contributions

CR participated in conceiving and designing the study, performed analyses with EC, interpreted the results, and drafted and revised the manuscript. EC participated in performing analyses. ER participated in conceiving the database and revising the manuscript. GK participated in revising the manuscript. IP participated in conceiving the database and in revising the manuscript. PC participated in conceiving the database, in interpreting the results and in revising the manuscript. All authors read and approved the final manuscript.

## Pre-publication history

The pre-publication history for this paper can be accessed here:

http://www.biomedcentral.com/1471-2458/11/949/prepub
